# Hibernation and Radioprotection: Gene Expression in the Liver and Testicle of Rats Irradiated under Synthetic Torpor

**DOI:** 10.3390/ijms20020352

**Published:** 2019-01-16

**Authors:** Walter Tinganelli, Timna Hitrec, Fabrizio Romani, Palma Simoniello, Fabio Squarcio, Agnese Stanzani, Emiliana Piscitiello, Valentina Marchesano, Marco Luppi, Maximiliano Sioli, Alexander Helm, Gaetano Compagnone, Alessio G. Morganti, Roberto Amici, Matteo Negrini, Antonio Zoccoli, Marco Durante, Matteo Cerri

**Affiliations:** 1 GSI Helmholtzzentrum für Schwerionenforschung, Biophysics Department, Planckstraße 1, 64291 Darmstadt, Germany; w.tinganelli@gsi.de (W.T.); a.helm@gsi.de (A.H.); 2 TIFPA, Trento Institute for Fundamentals Physics and Applications, Istituto Nazionale Fisica Nucleare, Via Sommarive 14-38123 Povo, TN, Italy; valentina.marchesano@tifpa.infn.it; 3 Department of Biomedical and Neuromotor Sciences, Physiology; Alma Mater Studiorum, University of Bologna, Piazza di Porta S.Donato, 2, 40126 Bologna, Italy; timna.hitrec@gmail.com (T.H.); fabio.squarcio@studio.unibo.it (F.S.); piscitiello.emiliana@gmail.com (E.P.); marco.luppi@unibo.it (M.L.); roberto.amici@unibo.it (R.A.); 4 Medical Physics, S.Orsola Malpighi University Hospital, Via Massarenti, 9-40138 Bologna, Italy; fabrizio.romani@aosp.bo.it (F.R.); gaetano.compagnone@aosp.bo.it (G.C.); 5 Department of Science and Technology, Parthenope University of Naples, Centro Direzionale isola C4, 80143 Napoli, Italy; palma.simoniello@uniparthenope.it; 6 Department of Veterinary Medical Sciences; Alma Mater Studiorum, University of Bologna, Via Tolara di Sopra, 50, Ozzano dell’Emilia, 40064 Bologna, Italy; agnese.stanzani2@unibo.it; 7 Istituto Nazionale Fisica Nucleare, Sezione di Bologna. Via Irnerio, 46, 40126 Bologna, Italy; Massimiliano.Sioli@bo.infn.it (M.S.); matteo.negrini@bo.infn.it (M.N.); Antonio.Zoccoli@bo.infn.it (A.Z.); 8 Department of Physics and Astronomy; Alma Mater Studiorum, University of Bologna, Via Irnerio, 46, 40126 Bologna, Italy; 9 Department of Experimental, Diagnostic and Specialty Medicine; Alma Mater Studiorum, University of Bologna, Via Massarenti, 9, 40138 Bologna, Italy; alessio.morganti2@unibo.it; 10 Technische Universität Darmstadt, Institut für Festkörperphysik, Hochschulstraße 6, 64289 Darmstadt, Germany

**Keywords:** radiation, liver, testicle, synthetic torpor, torpor, hypothermia, hibernation, raphe pallidus, ATM, space exploration

## Abstract

Hibernation has been proposed as a tool for human space travel. In recent years, a procedure to induce a metabolic state known as “synthetic torpor” in non-hibernating mammals was successfully developed. Synthetic torpor may not only be an efficient method to spare resources and reduce psychological problems in long-term exploratory-class missions, but may also represent a countermeasure against cosmic rays. Here we show the preliminary results from an experiment in rats exposed to ionizing radiation in normothermic conditions or synthetic torpor. Animals were irradiated with 3 Gy X-rays and organs were collected 4 h after exposure. Histological analysis of liver and testicle showed a reduced toxicity in animals irradiated in torpor compared to controls irradiated at normal temperature and metabolic activity. The expression of ataxia telangiectasia mutated (ATM) in the liver was significantly downregulated in the group of animal in synthetic torpor. In the testicle, more genes involved in the DNA damage signaling were downregulated during synthetic torpor. These data show for the first time that synthetic torpor is a radioprotector in non-hibernators, similarly to natural torpor in hibernating animals. Synthetic torpor can be an effective strategy to protect humans during long term space exploration of the solar system.

## 1. Introduction

Torpor is a peculiar state characterized by a drastic reduction in metabolic rate that leads to a decrease in body temperature proportional to the temperature gradient between the body and the environment [1]. Torpor is used by mammals as an energy saving strategy and can last from the few hours of an episode of daily torpor to many months during hibernation [2]. In terms of evolution, it is highly likely that torpor was a trait of the proto-mammal; therefore the gene set required to survive such state should reasonably be common among mammals [3,4].

Mimicking torpor in non-hibernators has always been an ambitious goal [5,6]. Indeed, such possibility would open interesting opportunity and could be very valuable in many clinical settings [7] but could also lead to applications in the field of space exploration [8]. To this last end, it is noteworthy that the European Space Agency (ESA) has a research group specifically focused on hibernation research for space exploration [9,10,11]. Besides the reduced need of energetic supply, torpor can in fact be extremely valuable for space exploration in virtue of the increased radioprotection that provides [12].

Biological damage from radiation is probably the most serious challenge that limits the ability for humans to engage in long term mission, because of the exposure to galactic cosmic radiation [13]. At the moment, shielding is not too effective against these kind of radiation, since mass constraints limits its use [14]. The Mars Science Laboratory measurements showed that the average dose-rate in deep space is about 1.8 mSv/day [15]. Therefore, a mission to Mars would exceed the career dose limit of 1 Sv of ESA astronauts and the gender- and age-specific limits for National Aeronautics and Space Administration (NASA) astronauts.

Hibernation would therefore be an effective solution for long term mission and was proposed to this end many years ago [16,17]. In fact, it was shown that hibernators are able to survive higher doses of radiation during torpor compared to the euthermic period [18]. This finding was confirmed for squirrels [19], hamsters [20] and mice [21]. The mechanism that mediates such enhanced radioprotection is not known; whereas earlier works were pointing out at the halt of cell replication, recent data suggest that the mechanism may lay in a different dynamics of the DNA damage and repair pathway [21,22].

Recently, a phenotype very much resembling torpor was induced in non-hibernators such as the rat by inhibiting the neurons within the region of the Raphe Pallidus (RPa) [23], a key brainstem region in the control of body temperature and energy expenditure in mammals [24]. It was proposed to call this peculiar state with the term “Synthetic Torpor” [7]. While the induction of synthetic torpor in humans has yet to be proven viable, such state was suggested as an effective way to transport animals in space both for scientific reason (i.e., experiments on the International Space Station or yet to come further permanent space stations) and for support a human colony [25]. However, so far, there is no evidence for the torpor-induced radioprotection to be active also during synthetic torpor induced in non-hibernators.

In this paper, we present preliminary data analysing gene expression in the liver and in the testis of rats which were irradiated during synthetic torpor compared with normothermic controls, showing that radioprotection is also induced during synthetic torpor and supporting the idea of using this state to preserve the health of humans and animals during long term space travels.

## 2. Results

**Figure 1:**
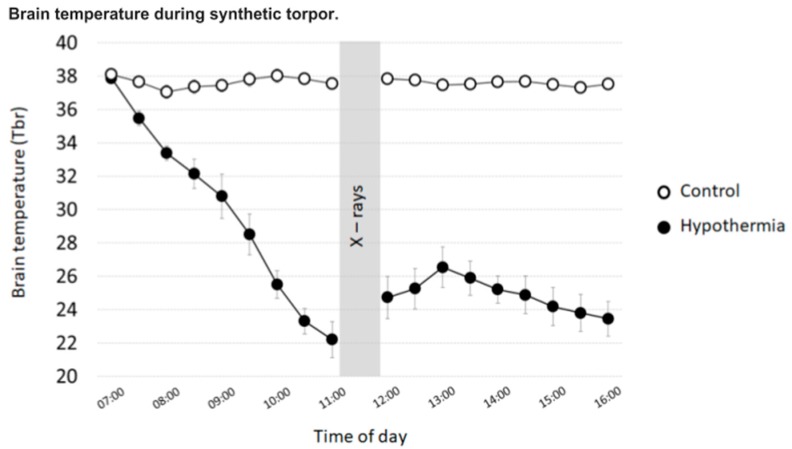
Thirty-min mean value ± SEM of the brain temperature (Tbr), recorded during the experimental day. Tbr was recorded by means of a thermistor, surgically implanted in the Lateral Hypothalamus. Empty dots: Control (*n* = 5), filled dots: Hypothermia (*n* = 5).

**Figure 2:**
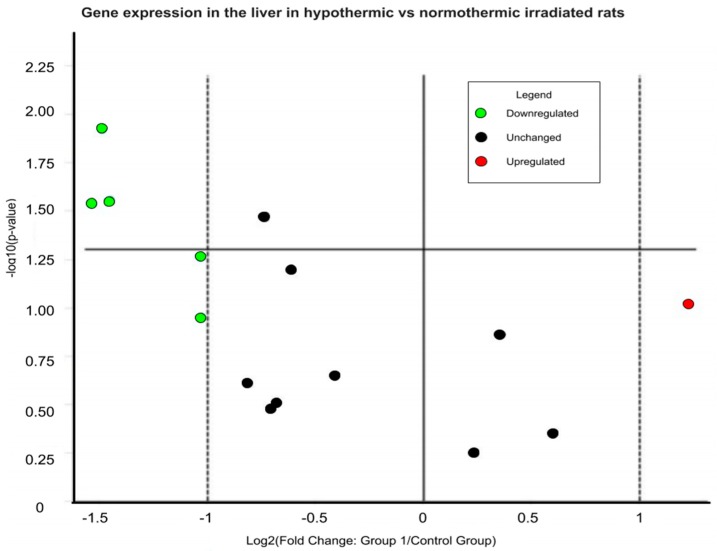
Volcano plot representing the expression of the analysed genes in the liver (normothermic vs hypothermic animals) 4 h after a total body irradiation (X-rays, 3Gy) expressed as normalized fold change. In green: genes downregulated (to the left of the left dashed line). In red: genes upregulated (to the right of the right dashed line). Dots above the solid horizontal line are significantly different between the two groups.

Rats induced into synthetic torpor showed a regular decrease in brain temperature (Tbr), which reached a nadir just before the irradiation (Figure 1) (Hypothermia, Tbr: 22.2 ± 1.1; Control, Tbr: 37.54 ± 0.24). Of the 12 genes analysed (Table S1), three genes were significantly downregulated in the Hypothermic group compared with the Control group: ATM (Fold regulation: −2.90; *p* = 0.02), Pfkb (Fold regulation: −2.73; *p* = 0.02) and Pdfe1α (Fold regulation: −2.80; *p* = 0.01) (Figure 2). ATM is of particular interest, since it is involved in repairing DNA double strand breaks. The lower expression of ATM in the group induced into synthetic torpor suggest that less damage was induced by radiation in the cell of the animals induced into synthetic torpor or that, for other reason, other DNA repair pathways become activated in such conditions.

**Figure 3:**
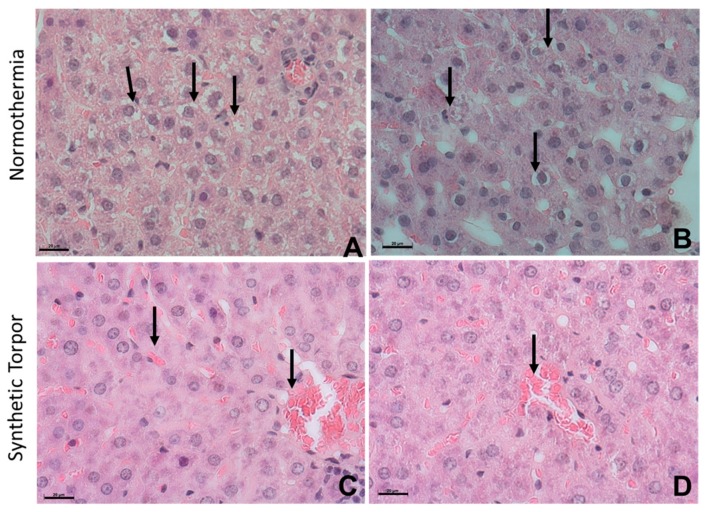
Morphology of the hepatic tissue after hematoxylin-eosin staining in irradiated normothermic liver (**A**,**B**) and in irradiated liver during synthetic torpor (**C**,**D**). The effects of irradiation at normothermia are pronounced compared to the effects detected after irradiation in synthetic torpor. Alteration and disorganization in the hepatic parenchyma were observed in **A**,**B**, vacuolization and shrunken nuclei with irregular shape and chromatin condensation (**A**,**B**, arrows) were identified in the hepatocytes. The morphology and the organization of hepatic parenchyma irradiated during synthetic torpor (**C**,**D**) seems comparable to the normal organization of the liver tissue. Hepatocytes show a dense cytoplasm, round nuclei with dispersed chromatin. An abundance of red blood cells is also observable (**C**,**D** arrows). Scale bars = 20 µm.

Qualitative analysis following the histological observation shows that hepatic tissues of normothermic rats were affected by the irradiation, whereas the hypometabolic-hypothermic state of synthetic torpor provide in the liver a protection to radiation damage. Normothermic rats showed altered morphology of the hepatocytes. At the light microscope level, their cytoplasm was low dense and markedly vacuolated, as well the nuclei appeared shrunken with irregular shape and chromatin condensation (Figure 3A,B, arrows). Such effects were not present in the hepatic tissue of the rats induced into synthetic torpor, hepatocytes possessed a dense cytoplasm and regular and round nucleus in which dispersed chromatin and nucleoli were detectable (Figure 3C,D). An abundance of red blood cells is also observable in the synthetic torpor group (Figure 3C,D, arrows).

**Figure 4:**
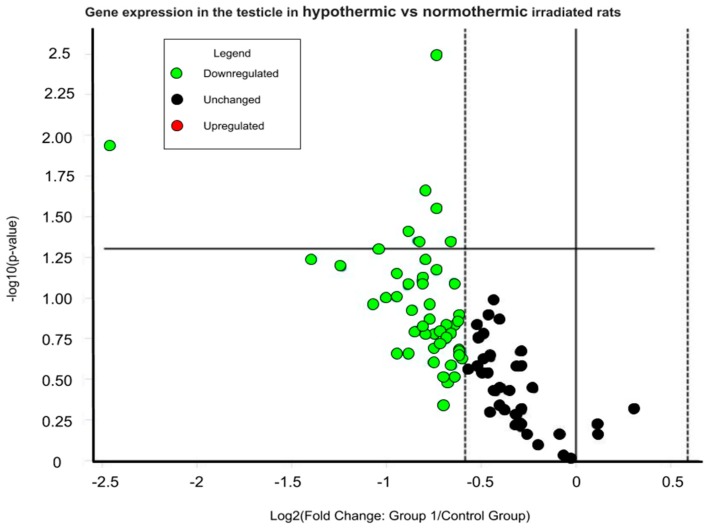
Volcano plot of the representing the expression of the analysed genes in the testicle (normothermic rat testis *vs* hypothermic rat testis) 4 h after a total body irradiation (X-rays, 3Gy) expressed as normalized fold change. In green: genes downregulated (to the left of the dashed line). Dots above the solid horizontal line are significantly different between the two groups.

A more extensive analysis of gene expression was performed on the testicles. Rats induced into synthetic torpor showed a downregulation of many of the genes involved in the DNA Damage Signaling. Of the 84 genes analyzed (Table S2), 7 genes were significantly downregulated in the Hypothermic group: Bbc3 (fold regulation: −5.49; *p* = 0.01), Fancg (fold regulation: −1.58; *p* = 0.04), Mbd4 (fold regulation: −1.79; *p* = 0.04), Mgmt (fold regulation: −1.84; *p* = 0.04), Pcna (fold regulation: −1.74; *p* = 0.02), Ppm1d (fold regulation: −1.67; *p* = 0.003), Rad18 (fold regulation: −1.66; *p* = 0.03) (Figure 4) (*p* values and fold regulation for all the genes analyzed are reported in Table S3).

**Figure 5:**
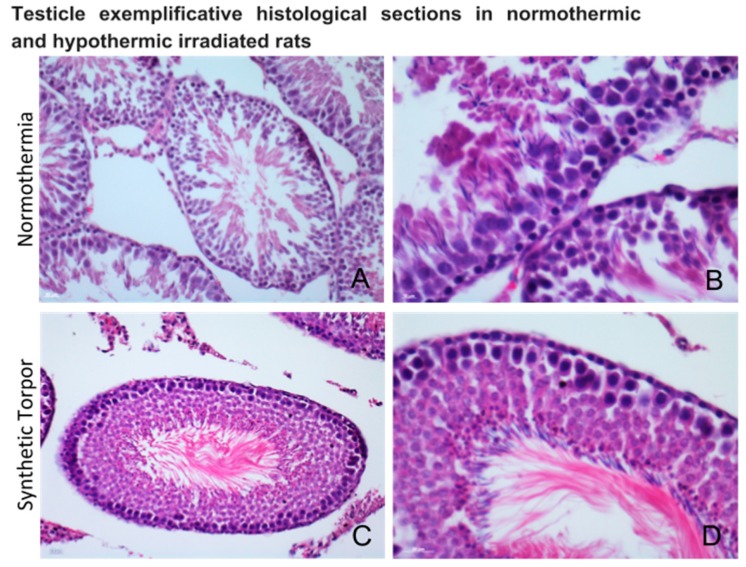
Morphology of cross sections of seminiferous tubules, after hematoxylin-eosin staining, in irradiated normothermic testis (**A**,**B**) and in irradiated testis during synthetic torpor (**C**,**D**). Alteration on the differentiation and cells stratification were found in the testis of normothermic rats (**A**,**B**), in particular disorganization of the germinal cells and of their junctions was present. These effects were not detected in the testis tissue of the rats induced into synthetic torpor, in which the structures could be comparable to the normal organization (**C**,**D**).

Histological analysis performed on cross sections of seminiferous tubules of testis shows alteration on the differentiation and cells stratification in the testis of normothermic rats (Figure 5A,B), in particular disorganization of the germinal cells and on their junctions were detected. These effects were not found in the testis tissue of the rats induced into synthetic torpor, in which the structures could be almost comparable to the normal organization (Figure 5C,D)

## 3. Discussion

The main finding reported in this paper is the significant protection to ionizing radiation damage induced by synthetic torpor in non-hibernating analysis. Histological analysis of liver and rats exposed to 3 Gy X-rays (see Figure 3 and Figure 5) show a reduced toxicity compared in animals artificially in torpor compared to controls irradiated at normal temperature and metabolic activity. This protection is consistent with old data in hibernating animals (reviewed in Reference [8]).

We have then investigated the molecular mechanisms underlying the increased radioresistance and found downregulation of ATM and several other genes involved in the DNA damage signalling. ATM expression was reported to be affected by deep hypothermia (0.8 °C) in cell culture [26,27] and we show here that synthetic torpor leads to similar effect in vivo.

ATM is a key gene in activating the repair process of DNA double strand breaks. The reduced expression of such gene suggest that the hepatocytes of the hypothermic group received less damage form the irradiation compared with the control group or that their DNA damage response is reduced.

Several papers suggested that the enhanced radioprotection that is observed during hypothermia both in vivo [21] or in vitro [22], may be caused by some change in the activation of the DNA repair pathways. In particular, data from Gosh and co-workers [21] show that the radioprotective effect can be induced by hypothermia within three hours after the irradiation. Such results apparently rules out other hypothesis that torpor-induced radioprotection could be caused by stopping cell proliferation or by reducing the oxygenation of tissues, even if these factors may certainly still play a role.

ATM and the DNA damage signaling genes are less activated either because cells in the hypothermic tissue received less DNA double strand breaks or because the reduced signaling cascade is part of the protective effect. It is however unlikely that the effect can simply be explained by hypothermia. In fact, the observed protection in genetic damage at low temperature has been associated to enhanced ATM activation [27], while we found a downregulation. On the other hand, ATM is the first post-irradiation protein to activate programmed cell death pathway [28]. In fact, pharmacological inhibition of ATM significantly reduces the radiosensitivity of unstimulated human lymphocytes [29]. The effect of synthetic torpor seems to be more similar to the pharmacological inhibition of ATM than to the simple hypothermia.

Histologically, liver cell of hypothermic rats appear normal, unlike the ones of the normothermic ones, suggesting that the radiation dosage was high enough to induce damage visible after a short time (4 h). Liver cells are extensively vacuolised, a sign that was reported to be a response to a stress agent [30,31]. Testicles also appear to be damaged by the irradiation in the normothermic group. In the present experiments only early normal tissue toxicity was evaluated. It is likely that late toxicity will also be strongly modified. ATM is involved in cell cycle progression post-irradiation [32] and the impact of irradiation during synthetic torpor on replication and late toxicity remain to be evaluated.

The reduction in metabolism in the synthetic torpor group did not suppress gene expression in general. In the liver, the gene Nfil3, for instance, results upregulated in this group. Such upregulation could be related to the involvement of Nfil3 in the negative feedback to the expression of the clock gene Per2, possibly adapting the clock to the reduced metabolism.

## 4. Methods

All the experiments were performed in accordance with the DL 26/2014 and the European Union Directive 2010/63/EU under the supervision of the Central Veterinary Service of the University of Bologna, under the approval of the Italian National Health Authority (decree 779/2017-PR, 16 October 2017).

### 4.1. Animal Housing

Experiments were conducted on 10 male Sprague-Dawley rats (Charles River), weighing 250–300 g. After their arrival, animals were housed for one week at standard laboratory conditions: light-dark (LD) cycle 12 h:12 h (L 09:00 h–21:00 h) with ad libitum access to food (4RF21 diet, Mucedola) and water, at an ambient temperature (Ta) of 24.0 ± 1.0 °C. During the adaptation, animals were housed in pairs in Plexiglas cages (Techniplast) containing dust-free wood shavings and the bedding was changed every two days.

### 4.2. Surgery

After one week of adaptation, rats underwent surgery under general anaesthesia (Ketamine-HCl, Imalgene 1000, Merial, 100 mg/Kg, intraperitoneally). After assessing the adequacy of the surgical plane of anaesthesia, animals were implanted with a thermistor probe in the right anterior hypothalamus to record deep brain temperature and with a microinjection guide cannula (C315G-SPC; Plastics One; internal cannula extension below guide: +3.5 mm) stereotactically implanted within the brainstem region of the Raphe Pallidus (RPa) (coordinates (mm) −3.4 posterior from the interaural, 0.0 Lateral, −9.5 from the brain surface, following the stereotactic map in reference [33]. To assess the correct positioning of the guide cannula, a functional intraoperative test was carried out: the GABA-A agonist muscimol (1 mM) was dissolved in artificial cerebrospinal fluid (ACSF) and injected (100nL) within the RPa and tail temperature was monitored through a temperature probe positioned on the tail. Previous reports showed that the inhibition of Raphe Pallidus neurons causes vasodilation [34]. The position of the cannula was then assumed correct if a consistent increase in tail surface temperature was observed within 5 min from muscimol injection. Lastly, four stainless steel screws were implanted, to ensure all the inserted probes in place; everything was then secured with dental resin (ResPal, Salmoiraghi Produzione Dentaria), covering the whole surgical field, incorporating the cannula, the thermistor and the screws. Rats were then administered with antibiotic (benzathine benzylpenicillin, 12.500.000 U.I., dihydrostreptomycin sulphate 5 g/100 mL, Rubrocillina Veterinaria, Intervet—1 mL/kg), analgesics (Carprofen—Rimadyl, Pfizer—5 mg/kg) and rehydrated with 5 mL saline solution. Animals were constantly monitored until regaining of consciousness and then left to recover for 1 week under standard laboratory conditions. The animal’s pain, distress or suffering symptoms were constantly evaluated using the Humane End Point (HEP) criteria. After at least one week of recovery from surgery, rats were moved to the experimental cages. The cage containing the animal was placed inside a thermoregulated, sound-attenuated box, equipped with a ventilation system. The cage was instrumented with a rotating swivel, connected to the external amplifiers. Forty-eight hours prior to the experiment, the animals’ thermistors were wired to the swivels, in order to acquire baseline deep brain temperature (Tbr) and animals were exposed to Ta 15 °C and constant darkness. Tbr) was amplified (mod. Grass 7P511L, Astronova, West Warwick, RI, USA) and filtered at 0.5 Hz (high-pass), analogue to digital converted to 12 bit (CED Micro MK 1401 II), acquired (50Hz sample rate) and stored on a digital hard disk.

### 4.3. Experimental Protocol

Animals were divided in two groups:

(1) Hypothermia (*n* = 5): After a 7-day recovery from surgery, animals were injected with the GABA-A agonist muscimol (1 mmoL) within the Raphe Pallidus (RPa). Each animal received, starting from 07:00 h, one injection/hour (100 nL). All animals entered synthetic torpor shortly after the first injection and maintained such condition until the end of the experiment.
(2) Control (*n* = 5): After a 7-day recovery from surgery, animals were injected with artificial cerebrospinal fluid (ACSF) within the RPa. Each animal received, starting from 07:00 h, one injection/hour (100 nL).

**Figure 6:**
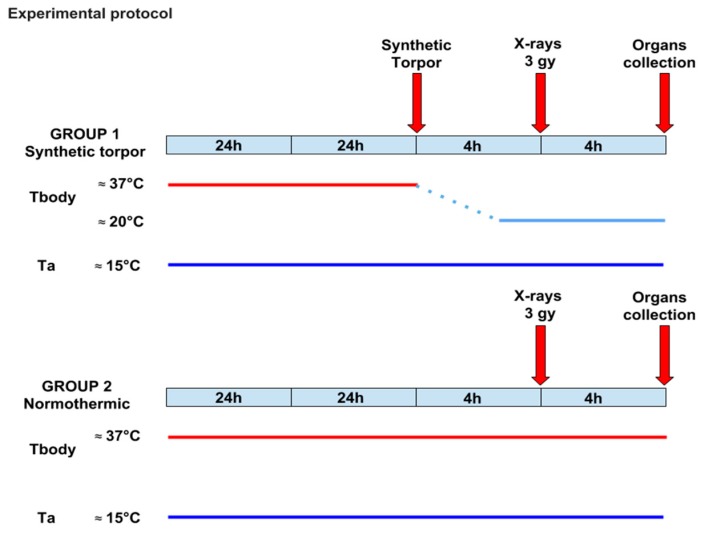
Experimental protocol. After at least one week of recovery from surgery, rats were moved to the experimental cage and exposed to a lower ambient temperature (15 °C) 48 h prior to the experiment. At 07:00 of the experimental day, rats were randomly assigned to one of the experimental groups: Hypothermia (*n* = 5) (Group 1), animals underwent multiple microinjections (1/h, 100 nL) of the GABA-A agonist muscimol (1mM) within the RPa, whereas Control (*n* = 5) (Group 2) were injected with artificial cerebrospinal fluid (ACSF). Four hours after the onset of synthetic torpor in the Hypothermia group, animals underwent a 3 Gy X-rays radiation exposure and were returned to the experimental cage for additional four hours. At 16:00, all animals were euthanized and organs were collected.

To minimize any environmental difference, animals underwent experiment in couples: each animal from the Hypothermia group underwent the procedure on the same experimental day as its corresponding Control. At 11:00 h, animals were moved to a portable thermoregulated and sound attenuated radiotransparent custom-made box, at Ta 15 °C. When inside the box, animals were transported to the Department of Nuclear Medicine, S.Orsola Hospital, to be X-rays irradiated. After receiving the radiation dose, animals were returned to the experimental cage. Four hours after the irradiation, animals were given a lethal dose of anaesthetic and several organs were extracted for histological and molecular assays (Figure 6).

### 4.4. Radiation Data

**Figure 7:**
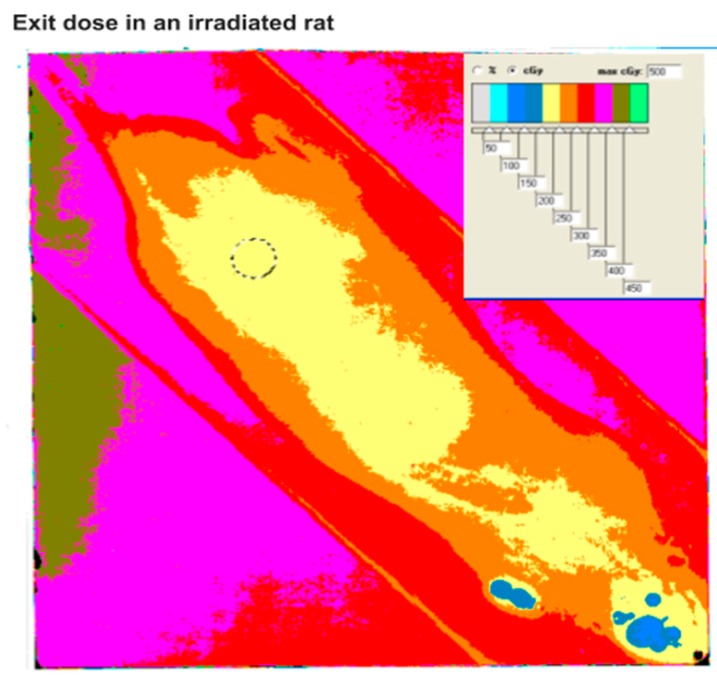
Exit dose measured with a radiochromic film under the irradiated animal. Entrance dose can be evaluated from the percentage depth dose curves and the animal thickness.

Exposure to radiation was accomplished by irradiating the thermoregulated transportable box from above, using an X-ray tube operating at 180 kV, producing a uniform radiation field in the whole region where the animal resides. Animals were irradiated with a dose of 3 Gy total body, at a dose rate of 23 cGy/min (Figure 7).

### 4.5. Gene Expression and Analysis

#### 4.5.1. Liver

Gene expression was assessed by a Custom RT2 Profiler PCR Arrays from Qiagen. In this analysis, 13 genes were profiled on 1 array in a biological triplicate. Each array also contains a panel of controls to monitor genomic DNA contamination (GDC) as well as controls for the first strand synthesis (RTC) and the real-time PCR efficiency (PPC). As a preliminary analysis, we analysed the expression of several genes involved in different pathways, from cell metabolism, to regulation of clock genes to the DNA damage pathway in order to have an overview of the cell functions (Table S1).

#### 4.5.2. Testicle

Gene expression was assessed by a DNA Damage Signaling RT2 Profiler PCR Arrays from Qiagen. In this analysis, 89 genes were profiled (Figure S2) on 3 array in a biological triplicate. The array contains also a panel of controls to monitor genomic DNA contamination (GDC) as well as controls for the first strand synthesis (RTC) and the real-time PCR efficiency (PPC).

The protocol used was the following:

The mature RNA was isolated from tissue (liver and testicles) using an RNA extraction kit (Qiagen RNeasy Plus Micro Kit, Hilden, Germany) according to the manufacturer’s instructions, and its quality was determined using a nanodrop (NanoDrop™ 2000 Spectrophotometers, Thermofisher). 1000 ng of RNA was used for the reverse transcription. It was reverse transcribed using a cDNA conversion kit (Qiagen QuantiTect Reverse Transcription Kit, Hilden, Germany). For the liver analysis, the cDNA in combination with RT2 SYBR^®^ Green qPCR Mastermix was used on a Custom RT2 Profiler PCR Array, while for the Rat testicles the gene expression was assessed using a real-time RT2 Profiler PCR Array (QIAGEN, Cat. no. PARN-029Z) in combination with RT2 SYBR^®^ Green qPCR Mastermix (Cat. no. 330529).

A CFX96 Biorad machine was used with the following cycles: Cycle 1; 10 min duration, 95 °C temperature; Cycle 40; 15 s duration and 95 °C temperature; 1 min duration, 60 °C temperature. CT values were exported to an Excel file to create a table of CT values. This table was then uploaded to the data analysis web portal at http://www.qiagen.com/geneglobe. Samples extracted from rats irradiated and not-hibernated, were assigned to the control group, whereas samples extracted from rats irradiated and hibernated were assigned to the test group. CT values were normalized based on a manual selection of reference genes.

The data analysis web portal calculated the fold change using the delta-delta CT method, in which the delta CT is calculated between gene of interest (GOI) and an average of housekeeping genes (HKG), followed by delta-delta CT calculations (delta CT (experiment)—delta CT (control)). The fold change is then calculated using the 2^(delta-delta CT)^ formula.

The *p* values were calculated based on a Student’s *t*-test of the replicate 2^(-Delta CT)^ values for each gene in the control group and treatment groups.

Gene expression data are presented with a Volcano Plot scheme. The volcano plot helps quickly identify significant gene expression changes. The volcano plot displays statistical significance versus fold-change on the *y*- and *x*-axes, respectively. The volcano plot combines a *p*-value statistical test with the fold regulation change enabling identification of genes with both large and small expression changes that are statistically significant.

#### 4.5.3. Histology

Liver and testicle samples were processed for histological investigations. Briefly, tissue samples were fixed in 4% PFA at 4 °C overnight, dehydrated in an ascending graded ethanol series, cleared with xylene for 30 min and embedded in paraffin at 58 °C. Sections (7 μm thick) were stained with haematoxylin–eosin to show general morphology.

## 5. Conclusions

This is the first experimental measurement of toxicity and gene expression in animals exposed to ionizing radiation under synthetic torpor. We have demonstrated that synthetic torpor increases radioresistance in non-hibernating animals (rats). The results indicate that synthetic torpor is a potential tool to enhance radioprotection in living organism during long term space mission.

## Supplementary Materials

Supplementary materials can be found at http://www.mdpi.com/1422-0067/20/2/352/s1.

## References

[1] Heldmaier G., Ortmann S., Elvert R. (2004). Natural hypometabolism during hibernation and daily torpor in mammals. Respir. Physiol. Neurobiol..

[2] Geiser F. (2004). Metabolic rate and body temperature reduction during hibernation and daily torpor. Annu. Rev. Physiol..

[3] Geiser F. (2008). Ontogeny and phylogeny of endothermy and torpor in mammals and birds. Comp. Biochem. Physiol. A Mol. Integr. Physiol..

[4] Malan A. (2014). The evolution of mammalian hibernation: Lessons from comparative acid-base physiology. Integr. Comp. Biol..

[5] Lee C.C. (2008). Is human hibernation possible?. Annu. Rev. Med..

[6] Bouma H.R., Verhaag E.M., Otis J.P., Heldmaier G., Swoap S.J., Strijkstra A.M., Henning R.H., Carey H.V. (2012). Induction of torpor: Mimicking natural metabolic suppression for biomedical applications. J. Cell. Physiol..

[7] Cerri M. (2017). The Central Control of Energy Expenditure: Exploiting Torpor for Medical Applications. Annu. Rev. Physiol..

[8] Cerri M., Tinganelli W., Negrini M., Helm A., Scifoni E., Tommasino F., Sioli M., Zoccoli A., Durante M. (2016). Hibernation for space travel: Impact on radioprotection. Life Sci. Space Res. (Amst.).

[9] ESA Advanced Concept Team. https://www.esa.int/gsp/ACT/projects/hibernation.html.

[10] Petit G., Koller D., Summerer L., Heldmaier G., Vyazovskiy V.V., Cerri M., Henning R.H., Seedhouse E., Shayler D. (2018). Hibernation and Torpor: Prospects for Human Spaceflight. Handbook of Life Support Systems for Spacecraft and Extraterrestrial Habitats.

[11] Gemignani J., Gheysens T., Summerer L. (2015). Beyond astronaut’s capabilities: The current state of the art. Conf. Proc. IEEE Eng. Med. Biol. Soc..

[12] Musacchia X.J., Barr R.E. (1968). Survival of whole-body-irradiated hibernating and active ground squirrels; Citellus tridecemlineatus. Radiat. Res..

[13] Durante M., Cucinotta F.A. (2008). Heavy ion carcinogenesis and human space exploration. Nat. Rev. Cancer.

[14] Durante M. (2014). Space radiation protection: Destination Mars. Life Sci. Space Res. (Amst.).

[15] Zeitlin C., Hassler D.M., Cucinotta F.A., Ehresmann B., Wimmer-Schweingruber R.F., Brinza D.E., Kang S., Weigle G., Bottcher S., Bohm E. (2013). Measurements of energetic particle radiation in transit to Mars on the Mars Science Laboratory. Science.

[16] Hock R.J. (1960). The potential application of hibernation to space travel. Aerosp. Med..

[17] Cockett T.K., Beehler C.C. (1962). Protective effects of hypothermia in exploration of space. JAMA.

[18] Jaroslow B.N., Smith D.E., Williams M., Tyler S.A. (1969). Survival of hibernating ground squirrels (Citellus tridecemlineatus) after single and fractionated doses of cobalt-60 gamma radiation. Radiat. Res..

[19] Barr R.E., Musacchia X.J. (1969). The effect of body temperature and postirradiation cold exposure on the radiation response of the hibernator Citellus tridecemlineatus. Radiat. Res..

[20] Musacchia X.J., Volkert W.A., Barr R.E. (1971). Radioresistance in hamsters during hypothermic depressed metabolism induced with helium and low temperatures. Radiat. Res..

[21] Ghosh S., Indracanti N., Joshi J., Ray J., Indraganti P.K. (2017). Pharmacologically induced reversible hypometabolic state mitigates radiation induced lethality in mice. Sci. Rep..

[22] Baird B.J., Dickey J.S., Nakamura A.J., Redon C.E., Parekh P., Griko Y.V., Aziz K., Georgakilas A.G., Bonner W.M., Martin O.A. (2011). Hypothermia postpones DNA damage repair in irradiated cells and protects against cell killing. Mutat. Res..

[23] Cerri M., Mastrotto M., Tupone D., Martelli D., Luppi M., Perez E., Zamboni G., Amici R. (2013). The inhibition of neurons in the central nervous pathways for thermoregulatory cold defense induces a suspended animation state in the rat. J. Neurosci..

[24] Morrison S.F., Madden C.J., Tupone D. (2014). Central neural regulation of brown adipose tissue thermogenesis and energy expenditure. Cell Metab..

[25] Griko Y., Regan M.D. (2018). Synthetic torpor: A method for safely and practically transporting experimental animals aboard spaceflight missions to deep space. Life Sci. Space Res. (Amst.).

[26] Dang L., Lisowska H., Manesh S.S., Sollazzo A., Deperas-Kaminska M., Staaf E., Haghdoost S., Brehwens K., Wojcik A. (2012). Effect of hypothermia on cells—A multiparametric approach to delineate the mechanisms. Int. J. Radiat. Biol..

[27] Lisowska H., Cheng L., Sollazzo A., Lundholm L., Wegierek-Ciuk A., Sommer S., Lankoff A., Wojcik A. (2018). Hypothermia modulates the DNA damage response to ionizing radiation in human peripheral blood lymphocytes. Int. J. Radiat. Biol..

[28] Herzog K.H., Chong M.J., Kapsetaki M., Morgan J.I., McKinnon P.J. (1998). Requirement for ATM in ionizing radiation-induced cell death in the developing central nervous system. Science.

[29] Heylmann D., Badura J., Becker H., Fahrer J., Kaina B. (2018). Sensitivity of CD3/CD28-stimulated versus non-stimulated lymphocytes to ionizing radiation and genotoxic anticancer drugs: Key role of ATM in the differential radiation response. Cell Death Dis..

[30] Chen N., Jiang J., Gao X., Li X., Zhang Y., Liu X., Yang H., Bing X., Zhang X. (2018). Histopathological analysis and the immune related gene expression profiles of mandarinfish (*Siniperca chuatsi*) infected with Aeromonas hydrophila. Fish Shellfish Immunol..

[31] Abdelhalim M.A., Jarrar B.M. (2012). Histological alterations in the liver of rats induced by different gold nanoparticle sizes, doses and exposure duration. J. Nanobiotechnol..

[32] Khoronenkova S.V., Dianov G.L. (2015). ATM prevents DSB formation by coordinating SSB repair and cell cycle progression. Proc. Natl. Acad. Sci. USA.

[33] Paxinos G., Watson C. (2007). The Rat Brain in Stereotaxic Coordinates.

[34] Nalivaiko E., Blessing W.W. (2001). Raphe region mediates changes in cutaneous vascular tone elicited by stimulation of amygdala and hypothalamus in rabbits. Brain Res..

