# Supplementary material for: Hibernation and Radioprotection: Gene Expression in the Liver and Testicle of Rats Irradiated under Synthetic Torpor
Source: Int J Mol Sci. 2019 Jan 16;20(2):352. doi: 10.3390/ijms20020352 (PMC6359347; doi:10.3390/ijms20020352)
Supplement: Supplementary file 1 [file ijms-20-00352-s001.pdf]

Supplementary material

List of analyzed genes in the liver

Symbol	Description
Hk1	Hexokinase 1
Nfil3	Nuclear factor, interleukin 3 regulated
Acsl1	Acyl-CoA synthetase long-chain family member 1
Pfkp	Phophofructokinase, platelet
Dsp	Desmoplakin
Akt1	V-akt murine thymoma viral oncogene homolog 1
Atm	Ataxia telangiectasia mutated homolog (human)
Id4	Inhibitor of DNA binding 4
Hp	Haptoglobin
Pde1alpha	Phosphodiesterase 1A, calmodulin-dependent
18SrRNA	Rat 18S rRNA sequence
Pgk1	Phosphoglycerate kinase 1
Actb	Actin, beta

Table S1 shows symbol and description of the analyzed genes in the liver

List of analyzed genes in the testicle

Symbol	Description
Abl1	C-abl oncogene 1, receptor tyrosine kinase
Apex1	APEX nuclease (multifunctional DNA repair enzyme)
Atm	Ataxia telangiectasia mutated homolog (human)
Atrx	Alpha thalassemia/mental ratardation syndrome X-linked (RAD54 homolog S. cerevisiae)
Bard1	BRCA1 associated RING domain 1
Bax	Bcl2-associated X protein

Bbc3	Bcl-2 binding component 3
Blm	Bloom syndrome, RecQ helicase-like
Brca1	Breast cancer 1
Brca2	Breast cancer 2
Cdc25a	Cell division cycle 25 homolog A (S. pombe)
Cdc25c	Cell division cycle 25 homolog C (S. pombe)
Cdkn1a	Cyclin-dependent kinase inhibitor 1A
Check1	CHK1 checkpoint homolog (S. pombe)
Check2	CHK2 checkpoint homolog (S. pombe)
Csnk2a2	Casein kinase 2, alpha prime polypeptide
Dclre1a	DNA cross-link repair 1A, PSO2 homolog (S. cerevisiae)
Ddb2	Damage specific DNA binding protein 2
Ddit3	DNA-damage inducible transcript 3
Ercc1	Excission repair cross-complementing rodent repair deficiency, complementation group 1
Ercc2	Excission repair cross-complementing rodent repair deficiency, complementation group 2
Exo1	Exonuclease 1
Fanca	Fanconi anemia, complementation group A
Fancc	Fanconi anemia, complementation group C
Fancd2	Fanconi anemia, complementation group D2
Fancg	Fanconi anemia, complementation group G
Fen1	Flap structure-specific endonuclease 1
Gadd45a	Growth arrest and DNA-damage-inducible, alpha
Gadd45g	Growth arrest and Dna-damage-inducible, gamma
Hus1	HUS1 checkpoint homolog (S. pombe)
Lig1	Ligase I, DNA, ATP-dependent

Mbd4	Methyl-CpG binding domani protein 4
Mgmt	O-6-methylguanine-DNA methyltransferase
Mif	Macrophage migration inhibitory factor
Mlh1	MutL homolog 1 (E. coli)
Mlh3	MutL homolog 3 (E. coli)
Mpg	N-methylpurine-DNA glycosylase
Mre11a	MRE11 meiotic recombination 11 homolog A (S. cerevisiae)
Msh2	MutS homolog 2 (E.coli)
Msh3	MutS homolog 3 (E.coli)
Nbn	Nibirin
Nthl1	Nth (endonuclease III)-like 1 (E.coli)
Ogg1	8-oxoguanine DNA glycosylase
Parp1	Poly (ADP-ribose) polymerase 1
Parp2	Poly (ADP-ribose) polymerase 2
Pcna	Proliferating cell nuclear antigen
Pms1	Postmeiotic segregation increased 1 (S. cerevisiae)
Pms2	PMS2 postmeiotic segregation increased 2 (S. cerevisiae)
Pold3	Polymerase (DNA-directed), delta 3, accessory subunit
Pole	Polymerase (DNA directed), epsilon
Polh	Polymerase (DNA directed), eta
Poli	Polymerase (DNA directed), iota
Ppm1d	Protein phosphatase 1D magnesium-dependent, delta isoform
Ppp1r15a	Protein phosphatase 1, regulatory (inhibitor) subunit 15A
Prkdc	Protein kinase, DNA activated, catalytic polypeptide
Pttg1	Pituitary tumor-transforming 1

Rad1	RAD1 homolog (S. pombe)
Rad17	RAD17 homolog (S. pombe)
Rad18	RAD18 homolog (S. cerevisiae)
Rad21	RAD21 homolog (S. pombe)
Rad50	RAD50 homolog (S. cerevisiae)
Rad51	RAD51 homolog (RecA homolog, E. coli) (S. cerevisiae)
Rad51c	RAD51 homolog c (S. cerevisiae)
Rad51b	RAD51 paralog b
Rad52	RAD52 homolog (S. cerevisiae)
Rad9	RAD9 homolog (S. pombe)
Rev1	REV1 homolog (S. cerevisiae)
Rnf8	Ring finger protein 8
Rpa1	Replication protein A1
Sirt1	Sirtuin (silent mating type information regulation 2 homolog) 1 (S. cerevisiae)
Smc1a	Structural maintenance of chromosome 1A
Smc3	Structural maintenance of chromosome 3
Sumo1	SMT3 suppresso of mif two 3 homolog 1 (S. cerevisiae)
Terf1	Telomeric repeat binding factor (NIMA-interacting)1
Topbp1	Topoisomerase (DNA) II binding protein 1
Tp53	Tumor protein 53
Tp53bp1	Tumor protein p53 binding protein 1
Ung	Uracil-DNA glycosylase
Wrn	Werner syndrome
Wrnip1	Werner helicase interacting protein 1
Xpc	Xeroderma pigmentosum, complementation group C

Xrcc1	X-ray repair complementing defective repair in Chinese hamster cells 1
Xrcc2	X-ray repair complementing defective repair in Chinese hamster cells 2
Xrcc6	X-ray repair complementing defective repair in Chinese hamster cells 6

Table S2 shows symbol and description of the analyzed genes in the testicle

P-value for all the genes analyzed in the testicle

Gene Symbol	Fold Regulation	p-value
Abl1	-1,54	0,1433477955
Apex1	-1,59	0,1517266476
Atm	-1,66	0,198130388
Atrx	-1,76	0,07908080364
Bax	-2,02	0,1008475313
Bbc3	-5,49	0,01158904208
Blm	-1,85	0,08506447535
Brca1	-1,75	0,1501535954
Brca2	-1,55	0,308506315
Cdc25a	-1,67	0,06892106106
Cdc25c	-1,75	0,07680293394
Cdkn1a	-2,07	0,05038942037
Chek2	-2,35	0,06520514199
Ddb2	-1,72	0,1693187276
Ddit3	-1,79	0,1578555593

Ercc2	-1,61	0,3048198355
Exo1	-1,58	0,1693315915
Fancc	-1,91	0,2189523619
Fancd2	-1,52	0,2356671832
Fancg	-1,58	0,044551593
Fen1	-1,57	0,2629629916
Gadd45g	-2,62	0,0579787066
Lig1	-1,94	0,09987136195
Mbd4	-1,79	0,04487391544
Mgmt	-1,84	0,0392603751
Mif	-1,76	0,1554792457
Mlh1	-2,11	0,1107374428
Mlh3	-1,93	0,07006498375
Mpg	-1,85	0,2260639129
Ogg1	-1,61	0,18214759
Pcna	-1,74	0,02224783331
Pms1	-1,73	0,1625826677
Pole	-1,67	0,1627865078
Polh	-1,81	0,1180838615
Ppm1d	-1,67	0,003227466353
Pttg1	-1,75	0,1589364943

Rad1	-1,53	0,1297668613
Rad17	-1,6	0,3320528412
Rad18	-1,66	0,02870144161
Rad51	-1,69	0,2503999896
Rad52	-1,53	0,2207906905
Rad9	-1,72	0,1343824981
Rev1	-1,64	0,161923206
Rnf8	-1,63	0,4552492587
Sirt1	-1,66	0,1658774743
Smc3	-1,74	0,05919226738
Ung	-1,77	0,156993114
Wrn	-1,56	0,0801427825
Xrcc1	-1,6	0,1468635898
Xrcc2	-1,52	0,2169630842
Xrcc6	-1,71	0,1128693361
Check1	-1,24	0,39552
Csnk2a2	-1,21	0,783206
Dclre1a	-1,31	0.608092
Ercc1	-1,4	0.377563
Fanca	-1,24	0.620050
Gadd45a	-1,43	0.253278
Hus1	-1,3	0.593755
Mre11a	-1,45	0.182590
Msh2	1,07	0.724642
Msh3	-1,24	0.358648

Nbn	1,23	0.506336
Nthl1	-1,35	0.481611
Parp1	-1,44	0.363357
Parp2	-1,34	0.246076
Pms2	-1,32	0.390605
Pold3	-1,36	0.166457
Poli	-1,33	0.501038
Ppp1r15a	-1,26	0.623647
Prkdc	-1,42	0.304382
Rad21	-1,23	0.746137
Rad50	-1,42	0.393958
Rad51c	-1,16	0.901608
Rad51b	-1,22	0.567819
Rpa1	-1,38	0.245941
Smc1a	-1,39	0.604607
Sumo1	1,08	0.756822
Terf1	-1,41	0.209488
Topbp1	-1,17	0.481340
Tp53	-1,05	0.995145
Tp53bp1	-1,49	0.366596
Wrnip1	-1,33	0.488990
Xpc	-1,26	0.720942

Table S3 shows the fold regulation and p value for all the genes analyzed in the testicle.
